# The Acute Cardiorespiratory and Cerebrovascular Response to Resistance Exercise

**DOI:** 10.1186/s40798-021-00314-w

**Published:** 2021-05-27

**Authors:** Blake G. Perry, Samuel J. E. Lucas

**Affiliations:** 1 School of Health Sciences, Massey University, Wellington, New Zealand; 2 School of Sport, Exercise and Rehabilitation Sciences & Centre for Human Brain Health, College of Life and Environmental Sciences, University of Birmingham, Birmingham, UK

**Keywords:** Resistance exercise, Cerebral blood flow, Blood pressure, Valsalva manoeuvre

## Abstract

Resistance exercise (RE) is a popular modality for the general population and athletes alike, due to the numerous benefits of regular participation. The acute response to dynamic RE is characterised by temporary and bidirectional physiological extremes, not typically seen in continuous aerobic exercise (e.g. cycling) and headlined by phasic perturbations in blood pressure that challenge cerebral blood flow (CBF) regulation. Cerebral autoregulation has been heavily scrutinised over the last decade with new data challenging the effectiveness of this intrinsic flow regulating mechanism, particularly to abrupt changes in blood pressure over the course of seconds (i.e. dynamic cerebral autoregulation), like those observed during RE. Acutely, RE can challenge CBF regulation, resulting in adverse responses (e.g. syncope). Compared with aerobic exercise, RE is relatively understudied, particularly high-intensity dynamic RE with a concurrent Valsalva manoeuvre (VM). However, the VM alone challenges CBF regulation and generates additional complexity when trying to dissociate the mechanisms underpinning the circulatory response to RE. Given the disparate circulatory response between aerobic and RE, primarily the blood pressure profiles, regulation of CBF is ostensibly different. In this review, we summarise current literature and highlight the acute physiological responses to RE, with a focus on the cerebral circulation.

## Key Points

- Dynamic resistance exercise produces a profoundly different haemodynamic response to aerobic type exercise, including the cerebral blood flow profile. Therefore, the relative contribution of regulatory mechanisms governing cerebral blood flow likely differs greatly between exercise types. Specificity is required when describing exercise to elucidate the underlying physiological mechanisms/responses.
- The type of muscular contraction dictates the blood pressure and cerebral blood flow profile during resistance exercise, further complicated by recruitment of the Valsalva manoeuvre. The Valsalva manoeuvre exacerbates the within-exercise blood pressure, although is associated with acute reductions in cerebral blood flow and increased risk of post-exercise syncope.
- The ability to accurately measure cerebral blood flow during exercise is problematic, restricting investigations to certain exercises and precludes the recruitment of the Valsalva manoeuvre.

## Introduction

Resistance exercise (RE) produces many favourable physiological outcomes that include increased muscular strength, metabolism and alterations in lean body mass [1]. Recently, RE has gained in popularity and is considered an integral part of general health and wellbeing, countering the age-related decline in muscle mass [2] and recommended in combination with regular aerobic exercise to maintain physical function during ageing [3]. Furthermore, RE appears neuroprotective, with transient increases in brain-derived neutrophic factor, a key biomarker linked to neurogenesis and neuronal survival [4], reported immediately following exercise cessation [5, 6]. More recently, RE is recommended as treatment for clinical cohorts, including type II diabetes mellitus [7, 8], stroke [9] and heart disease [10, 11]. Although rare, the acute extreme hypertension experienced during RE has potential to produce cerebrovascular injury [12, 13], with intense physical activity and the Valsalva manoeuvre (VM) identified as independent triggers of intracranial aneurysm rupture [14]. More common is the risk of syncope immediately following exercise cessation [15], which has the potential to cause trauma related injury. Nevertheless, the benefits of RE are vast and regular participation should be promoted to support physical wellbeing.

The term RE encompasses multiple variations of generating muscular force against an external load and can be subdivided into static and dynamic exercise. The terms, static and dynamic, refer to the type of muscular contraction performed. Static refers to an isometric contraction, sustained for a given period (e.g. 15 s); whilst dynamic generally refers to a repetitive movement cycle that consists of distinct concentric and eccentric phases (changes in muscle length), with an associated change in joint angle. Each complete cycle is termed a *repetition*, with a *set* comprising of a given number of repetitions. In addition, some research has utilised rhythmic exercise, consisting of static contractions interspersed with relaxation of varying duty cycles. The distinction between the types of RE dictates the haemodynamic response, with key differences discussed herein.

## Review Scope

The review will focus primarily on data from healthy and young (<40 years of age) participants. The review will first briefly cover cerebrovascular regulation to contextualise the regulatory mechanisms that will be active during RE. From here, the review is divided into three main sections. The first section will discuss the physiological response to the VM in isolation and in relation to RE. The second section will discuss the haemodynamic response *during* RE; including differences between exercise type (static versus dynamic) and the effects of exercise intensity. The final section covers the response immediately *following* RE. Methodology regarding the measurement of cerebral blood flow (CBF) is also considered.

## Regulation of Cerebral Blood Flow

The brain displays differential regulation compared to other vascular beds. It is immensely sensitive to carbon dioxide (CO_2_), far more so than the skeletal muscle [16], and the parenchyma is extremely ischemia intolerant, with limited ability for metabolic substrate storage and an extraordinarily high metabolic rate [17]. As such, CBF is under fine control at rest, and exercise, modulated by a plethora of variables that include neurovascular coupling (NVC) [18], cerebral perfusion pressure (CPP) [19], humoral factors (e.g. partial pressure of arterial CO_2_ and O_2_) [20, 21], cardiac output [22] and the autonomic nervous system [23–25].

At rest, the partial pressure of arterial carbon dioxide (P_a_CO_2_) appears to be the most potent and dominating regulator of CBF [26]. Reductions in P_a_CO_2_ (hypocapnia) stimulate vasoconstriction of cerebral arterioles, increasing cerebrovascular resistance and ultimately reducing CBF [27]. Conversely, increases in P_a_CO_2_ (hypercapnia) vasodilate cerebral arterioles, reducing cerebrovascular resistance and elevating CBF [28, 29]. The regulatory hierarchy persists during exercise, and during aerobic exercise, the CBF profile resembles an inverted U, initial increases to moderate intensities (50–80% of maximal workload) with hypocapnic-induced reductions evident at maximal exercise [30]; albeit mostly studied in cycling-based aerobic exercise.

Whilst the current body of knowledge describes the role of CO_2_ in cerebral perfusion comprehensively, the regulation of CBF during changes in CPP has not been as straightforward. Cerebral autoregulation refers to the process by which CBF is held relatively constant despite varying perfusion pressure [31]. When CPP increases cerebral vessels vasoconstrict, limiting hyperperfusion, conversely, when CPP is decreased cerebral vessels dilate to maintain CBF [17, 32]. The effectiveness of cerebral autoregulation has been recently challenged [33, 34], with the autoregulatory plateau much narrower than previously described [35, 36]. This is pertinent given the hallmark of dynamic RE is large sinusoidal changes in mean arterial pressure (MAP). Furthermore, the modulators of CBF described above are intertwined and not active in isolation. This is exemplified by the interaction between cerebral autoregulation and P_a_CO_2_, whereby hypocapnia improves cerebral autoregulation while hypercapnia impairs it [19]. Discussion of CBF modulators in detail is beyond the scope of this review, and the reader is directed to more comprehensive reviews of CBF regulation at rest [17, 32, 37, 38] and exercise [30]. Nevertheless, there are some unique aspects of RE that need to be considered in the context of CBF regulation.

## The Valsalva Manoeuvre

**Fig. 1:**
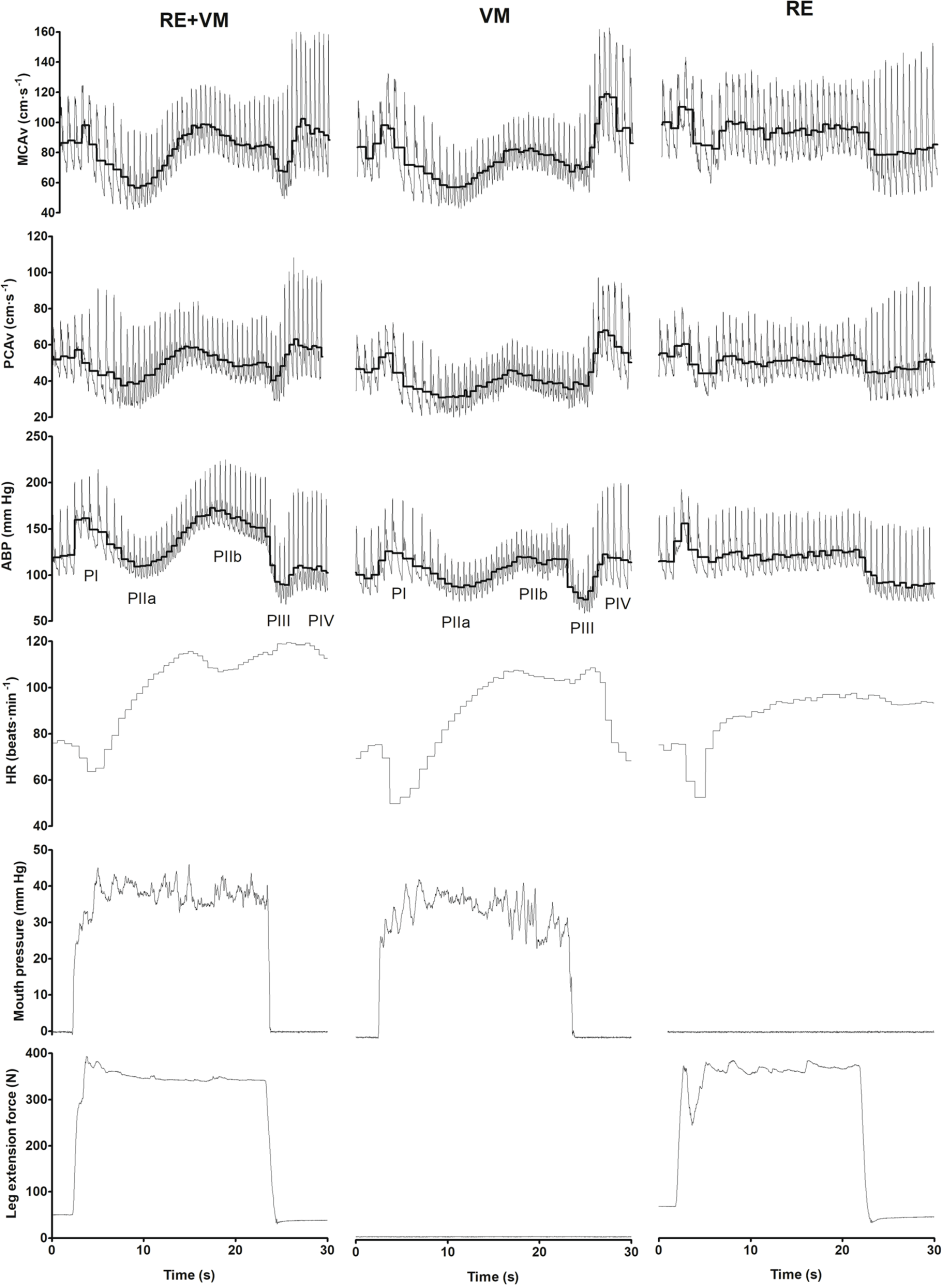
Typical trace for a combination of RE and VM (RE + VM), Valsalva manoeuvre (VM) in isolation, and isometric RE in isolation (RE). Phases of the VM (phase I (PI) through phase 4 (PIV)) are visible in both RE+VM and VM conditions. The thick black line in middle cerebral artery blood velocity (MCAv), posterior cerebral artery blood velocity (PCAv) and arterial blood pressure (ABP) traces represents the mean value for each cardiac cycle. HR heart rate. Reproduced with permission from [45]

Traditionally, the VM is performed by forceful exhalation against a closed glottis and used in everyday activities such as lifting [39], defecation and coughing [40]. In isolation the VM produces a complex cardiovascular response [41], used clinically to test dynamic cerebral autoregulation [42, 43] and autonomic function [44]. There are 4 distinct phases of the VM (see Fig. 1). The first (phase I) is characterised by a transient MAP spike as increased intrathoracic pressure is translated to the arterial tree [40]. Approximately 3 s into the strain, atrial filling (phase IIa) declines [39, 46], reducing stroke volume (SV) and subsequently MAP. Arterial baroreflex-mediated increases in heart rate (phase IIb) generate a partial recovery of MAP until the strain is released. Upon strain cessation, blood floods the distended pulmonary vessels and the reduction in intrathoracic pressure decompresses the thoracic arteries, reducing MAP (phase III), the magnitude dependent upon straining intensity [47] and body position [42]. The now elevated cardiac output is ejected against a constricted systemic circulation, a remnant of the vascular arm of the baroreflex response from preceding phases, transiently increasing MAP (phase IV) [42, 43, 48]. A more detailed account of the physiology of an isolated VM can be found elsewhere [41].

### Cerebrovascular Response to the Valsalva Manoeuvre

As mentioned, CBF is regulated by a myriad of physiological variables, many of which undergo rapid and bidirectional perturbations during the VM alone. A hallmark of the VM is rapid changes in CPP, and although cerebral autoregulatory mechanisms are active, they are unable to effectively counter such changes [43, 49]. CPP is calculated by subtracting intracranial pressure (ICP) from MAP, and as ICP is elevated during a VM [40, 50], significant reductions in CPP can quickly ensue, particularly during phases IIa-IIb when MAP is reduced. The VM also elevates central venous pressure (CVP) [42], which restricts cerebral venous outflow, raises intracranial blood volume and subsequently ICP [51]. The increase in CVP may exceed that of CSF pressure and become the major determinant of ICP during the VM, with elevations in CVP reducing CBF [52, 53]. Without an obvious VM, changes in intrathoracic pressure may persist during RE and alter cerebral venous drainage [54], particularly during axial loading of the spine as seen during upright squatting [55].

**Fig. 2:**
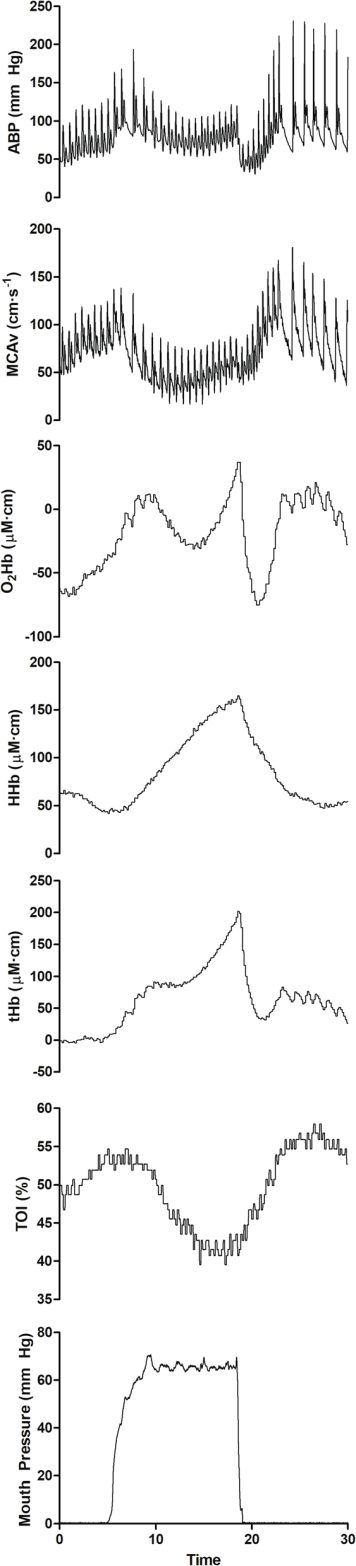
Hemodynamic variables in one participant during a Valsalva manoeuvre at 90% of maximal mouth pressure for 10s. ABP arterial blood pressure; MCAv_mean_ mean middle cerebral artery blood velocity; TOI total oxygenation index; O_2_Hb oxyhaemoglobin; HHb deoxyhaemoglobin; tHb total haemoglobin. Reproduced with permission from [57]

MAP increases during phase I, with no change [47, 56] or a modest increase [42, 57] in middle cerebral artery blood velocity (MCAv) reported. Furthermore, greater strains, despite producing greater hypertension, do not produce a greater increase in MCAv during phase 1 [47]. One proposed explanation is that ICP elevations mechanically restrain MCAv during phase I. Whilst the exact role of the autonomic nervous system in CBF control remains controversial [58], sympathetic vasoconstriction may have a similar function during high CPPs [59–61] and limit hyperperfusion. Comparatively, the phase IV response, associated with a normalisation of ICP and rapid concurrent increases in MAP, produces a greater peak MCAv response than phase I [56]. In addition to phase IV reductions in ICP, functional hyperaemia contributes to the observed cerebral hyperperfusion [57], driven by an acute reduction in cerebral oxygenation during phase II (Fig. 2), compounded by an elevated P_a_CO_2_ [42] and a cerebral autoregulatory vasodilation persisting from phase III [43].

It should be noted most of the data concerning the VM, and data comprising this review, are from males (see Tables 1 and 2). This is problematic given that the phase IIb response to the VM is sex-dependent, with smaller increases in mean and diastolic MCAv observed in men [84]. Women also have a greater resting MCAv [85, 86] and differential cerebrovascular reactivity to CO_2_ [87, 88]. Additionally, cerebral autoregulation has been shown to be greater in the anterior cerebral artery [85] and MCA [89] vascular territories during acute hypotension in females, which persists irrespective of menstrual cycle phase [90]. As such, extrapolation of the discussed response to females is cautioned and further research is required to quantify the haemodynamic response to RE in females.

**Table 1:** Studies investigating CBF responses to dynamic resistance exercise

	Intensity	Exercise performed	Cohort	Contraction number and duty cycle	CBF metrics	VM	CBF response	MAP response
Dickerman et al. [62]	100% of 1RM	Bilateral leg press	**Mean age:** 31 ***n*:** 9 Healthy resistance trained males	**Duty cycle:** Not stated **Number:** 1	Unilateral MCAv – averaged across exercise	Yes	Mean decrease in MCAv_mean_ of 16 cm^.^s^-1^. Unclear if pre exercise hyperventilation contributed as neither P_ET_CO_2_ or P_a_CO_2_ were reported	Not reported
Edwards et al. [12]	Self-selected 10RM, equates to ~75% of MVC	Bilateral leg press	**Mean age:** 21 ***n*:** 9 Healthy resistance trained participants (6 females)	**Duty cycle:** not stated **Number:** 10	Unilateral MCAv – averaged across exercise	No	No change during exercise but acute reduction in MCAv_mean_ (mean 7 cm^.^s^-1^) below rest within 5s of exercise cessation	Mean increase of ~15 mm HG in MAP during exercise with no post exercise hypotension reported.
Koch et al. [63]	Endurance: 50–60% of maximum Strength: 80–90% of maximum	Bilateral leg extension	**Mean age:** males = 25, females = 24 ***n*:** 39 Healthy resistance trained participants (17 females)	**Duty cycle:** ~2s contraction time **Number:** Until exhaustion. Endurance maximum of 23 repetitions and strength maximum of 8 repetitions.	Bilateral MCAv	No – but brief VM could not be excluded.	15–30% increases in MCAv_mean_ in both intensities examined. Temporarily impaired autoregulation following exercise with evidence of presyncopal reactions.	Increase at both intensities and rapid decline following exercise.
Moralez et al. [64]	Estimated 10RM	Bilateral leg press exercise	**Mean age:** 24 ***n*:** 10 Healthy males	**Duty cycle:** 10 repetitions per 30s (0.33hz) **Number:** 10 Repetitions	Unilateral MCAv – averaged across exercise	No	Increase in MCAv_mean_. Acute reduction when standing immediately following exercise	Increase in MAP. Acute reduction upon standing immediately following exercise
Perry et al. [55]	30, 60 and 90% of 6RM	Upright squatting	**Mean age:** 26 ***n*:** 12 Healthy resistance trained males	**Duty cycle:** 2s eccentric and 2s concentric **Number:** 2 and 6 repetitions	Unilateral MCAv. Peak MCAv (at onset of concentric phase) analysed per repetition and absolute decrease following exercise	Yes, only observed at 90% 6RM	Similar mean increases of 31% in peak MCAv_mean_ across all intensities. Larger decrease in MCAv_mean_ post exercise at 90% 6RM.	Intensity dependent increase in MAP during exercise and reduction immediately following exercise.
Romero and Cooke [65]	80% of 6RM	Bilateral leg press	**Mean age:** 26 ***n*:** 10 Healthy resistance trained participants (5 females)	**Duty cycle:** Not stated **Number:** 10 Repetitions	Unilateral MCAv – averaged across exercise	No	12% increase in MCAv_mean_ without pre-exercise hyperventilation. Acute reduction when standing immediately following exercise. Pre exercise hyperventilation reduced MCAv during exercise.	Increase in MAP. Acute reduction upon standing immediately following exercise

*MVC* maximal voluntary contraction, *MCAv* middle cerebral artery blood velocity, *PCAv* posterior cerebral artery blood velocity, *P*_*ET*_*CO*_*2*_ partial pressure of end-tidal carbon dioxide, *P*_*a*_*CO*_*2*_ partial pressure of arterial carbon dioxide, *MAP* mean arterial blood pressure, *RM* repetition maximum, *VA* vertebral artery, *CBF* cerebral blood flow, *VM* Valsalva manoeuvre

**Table 2:** Studies investigating CBF responses to static and rhythmic resistance exercise

	Type of resistance exercise and intensity	Exercise performed	Cohort	Contraction Variables	CBF metrics	VM	CBF response	MAP response
Braz et al. [66]	**Type:** Static **Intensity:** 40% of MVC	Unilateral handgrip	**Mean age:** 20 ***n*:** 10 Healthy males	**Duration:** Until task failure **Number:** 1	Contralateral MCAv	No	Increase in MCAv_mean_ only when P_ET_CO_2_ was clamped 1 mm Hg above resting. No change during control	Gradual increase up to task failure
Fernandes et al. [67]	**Type:** Static **Intensity:** 30% MVC	Unilateral hand grip	**Mean age:** 27 ***n*:** 9 Healthy recreationally active males	**Duration:** 2 min **Number:** 1	Bilateral ICA blood flow. Average across last 30s of contraction.	No	Increase in Contralateral ICA blood flow only	Elevated from baseline
Friedman et al. [68]	**Type:** Static **Intensity:** 10 and 20% of MVC.	Unilateral handgrip	**Mean age:** 30 ***n*:** 8 Healthy participants (2 females)	**Duration:** 4.5 min **Number:** 3	Regional and hemispheric CBF via Xenon inhalation with rotating single photon tomograph	No	No change in hemispheric CBF. Increase in premotor and motor sensory blood flow bilaterally.	Mean ~7 mm and 14 mm Hg increase during 10% and 20% MVC, respectively
Giller et al. [69]	**Type:** Rhythmic **Intensity:** volitional maximum	Unilateral handgrip	**Mean age:** 34 ***n*:** 20 Healthy participants (7 females)	**Duty cycle:** 1Hz **Number:** Continuous for 5 min	Bilateral MCAv – averaged over the last 2 minutes of exercise	No	Bilateral increase in MCAv_mean_–Mean increase of 13% and 10% for contralateral and ipsilateral MCAv_mean_ respectively	24% increase
Hartwich et al. [70]	**Type:** Rhythmic **Intensity:** 10, 25 and 40% of MVC	Unilateral hand grip	**Mean age:** 22 ***n*:** 9 Healthy recreationally active participants (1 female)	**Duration:** 7 min **Duty cycle:** 1s contraction - 2s relaxation	Contralateral MCAv	No	No change across all intensities investigated	No change across all intensities investigated
Hirasawa et al. [71]	**Type:** Static **Intensity:** 30% of MVC	Unilateral leg extension	**Mean age:** 21 ***n:*** 12 Healthy Participants (8 females)	**Duration:** 2 min **Number:** 1	Contralateral ICA blood flow and MCAv, ipsilateral ECA blood flow. Measured in 30s bins	No	Increased ICA flow throughout contraction. MCAv_mean_ increased from 60s and was maintained	Gradual increase and plateaus after 90s.
Imms et al. [72]	**Type:** Static **Intensity:** 40% MVC	Unilateral handgrip	**Age range:** 18-38 ***n:*** 27 Healthy participants (6 females)	**Duration:** 2 min **Number:** 1	Contralateral MCAv	No	Increase in MCAv_mean_ by 17.5% in participants that did not hyperventilate. Participants that hyperventilated and reduced P_ET_CO_2_ by 8–15 mm Hg showed a non-significant increase in MCAv_mean_ of ~2 cm^.^s^-1^	Mean increase of 39 mm Hg
Ide et al. [73]	**Type:** Rhythmic **Intensity:** 20% MVC	Unilateral handgrip	**Mean age:** 31 ***n:*** 9 Sex and training status not reported	**Duty cycle:** 1 Hz **Duration:** 5 min	Bilateral MCAv	No	Contralateral increase in MCAv_mean_ of 13% with a smaller 6% increase on the ipsilateral side	12 mm Hg increase
Jørgensen et al. [74]	**Type:** Rhythmic **Intensity:** Not specified.	Unilateral handgrip	**Mean age:** 27 ***n:*** 12 (7 females)	**Duty cycle:** 30 contractions per minute **Duration:** 5 min	Bilateral MCAv, sampled every 30s	No	20% and 24% increase in contralateral MCAv_mean_ during right and left hand contractions respectively. No change in ipsilateral MCAv_mean_ observed in either conditions.	20 mm Hg increase in
Jørgensen et al. [75]	**Type:** Static **Intensity:** 30% of MVC.	Unilateral Knee extension	**Median age:** 33 ***n:*** 11 (2 females)	**Duration:** 5 min **Number:** 1	Bilateral MCAv—data collected each minute over exercise. Xenon clearance technique and measured during 3 minutes of exercise.	No	No change in MCAv_mean_ or CBF in either hemisphere.	16 mm Hg increase during exercise
Kim et al. [76]	**Type:** Rhythmic **Intensity:** 65% of MVC	Unilateral handgrip	**Mean age:** 25 ***n:*** 7 Healthy recreationally active males	**Duty cycle:** 2s contraction with 4s rest **Number:** continuous for10 min	Contralateral MCAv	No	Maintained increase in MCAv_mean_ at 5 and 10 minutes during exercise	Sustained ~20% increase in MAP throughout exercise
Linkis et al. [77]	**Type:** Rhythmic **Intensity:** Not specified for handgrip. Load of 4.8kg for foot movements	Unilateral handgrip and foot movements	**Mean age:** 26 ***n:*** 14 (6 females)	**Duty cycle:** 1 Hz **Duration:** 15 min	Bilateral MCAv and ACAv	No	19% increase in contralateral MCAv_mean_ during hand contractions. 23% increase in contralateral ACAv_mean_ during foot movements and 11% increase in ipsilateral MCAv_mean and_ ACAv_mean_	17 mm Hg increase in MAP during hand contractions. 10 mm Hg increase during foot movements
Ogoh et al. [78]	**Type:** Static **Intensity:** 30% of MVC.	Unilateral handgrip	**Mean age:** 22 ***n:*** 9 Healthy participants (4 females)	**Duration:** 2 min **Number:** 1	Ipsilateral MCAv	No	Mean 9 cm^.^s^-1^ increase in MCAv. Static resistance exercise did not modify dynamic cerebral autoregulation	Mean 16 mm Hg increase
Pott et al. [79]	**Type:** Static **Intensity:** 100% of MVC	Bilateral leg extension	**Mean age:** 28 ***n:*** 10 Healthy participants (4 females)	**Duration:** 15s **Number:** 2	Unilateral MCAv and tissue oxygenation via NIRS	One bout with normal ventilation and one bout with a VM	Dependent upon VM recruitment. With continued ventilation MCAv_mean_ increased initially and then declined to baseline values.	Lower MAP when ventilation was maintained.
Perry et al. [45]	**Type:** Static **Intensity:** 50% of MVC.	Bilateral leg extension	**Mean age:** 28 ***n*:** 11 Healthy recreationally active participants (2 females)	**Duration:** 15s **Number:** 2	MCAv, PCAv and VA blood flow	One bout with normal ventilation and one bout with a VM	Larger initial increase in MCAv during exercise without a VM. Both MCAv and PCAv elevated throughout exercise. No difference in VA blood flow between re with and without VM.	No initial difference in MAP increase at exercise onset with and without VM. After ~10s MAP is significantly greater with concurrent VM
Vianna et al. [80]	**Type:** Static and rhythmic **Intensity:** 35% of MVC	Unilateral calf exercise (plantarflexion)	**Mean age:** 24 ***n*:** 16 Healthy participants (4 females)	**Duty cycle:** rhythmic 0.5s contraction, 0.5s relaxation **Number:** not stated	Contralateral ACAv	No	Similar mean increase in ACAv_mean_ of 15% during static and rhythmic	Similar increases in MAP during both types of exercise
Washio et al. [81]	**Type:** Static **Intensity:** 30% of MVC.	Unilateral handgrip	**Mean age of entire cohort:** 21 ***n*:** 11 Healthy male participants	**Duration:** until exhaustion (<90% of workload) **Number:** 1	Ipsilateral PCAv and VA blood flow from various sides. Averaged over the last 30s of exercise.	No	Mean ~3 cm^.^s^-1^ increase in PCAv. Mean ~ 38 ml^.^min^-1^ increase in VA blood flow	Mean ~25 mm Hg increase
Washio et al. [82]	**Type:** Static **Intensity:** 30% of MVC.	Unilateral handgrip	**Mean age:** 25 ***n*:** 9 Healthy male participants	**Duration:** 3 min **Number:** 1	Contralateral MCAv and ipsilateral VA blood flow. Averaged over the last 30s of exercise.	No	No change in MCAv. Mean ~35 ml^.^min^-1^ increase in VA blood flow	Non-significant mean increase of 28 mm Hg
Yamaguchi Et al. [83]	**Type:** Static **Intensity:** 30% of MVC.	Unilateral handgrip	**Mean age:** 25 ***n*:** 17 Healthy male participants	**Duration:** 2 min **Number:** 1	Contralateral PCAv	No	Mean 4 cm^.^s^-1^ increase in PCAv.	Mean ~19 mm Hg increase

*MVC* maximal voluntary contraction, *ACAv* anterior cerebral artery blood velocity, *MCAv* middle cerebral artery blood velocity, *PCAv* posterior cerebral artery blood velocity, *P*_*ET*_*CO*_*2*_ partial pressure of end-tidal carbon dioxide, *MAP* mean arterial blood pressure, *RM* repetition maximum, *VA* vertebral artery, *ICA* internal carotid artery, *ECA* external carotid blood flow, *CBF* cerebral blood flow, *VM* Valsalva manoeuvre

### Role of the Valsalva Manoeuvre During Resistance Exercise

**Fig. 3:**
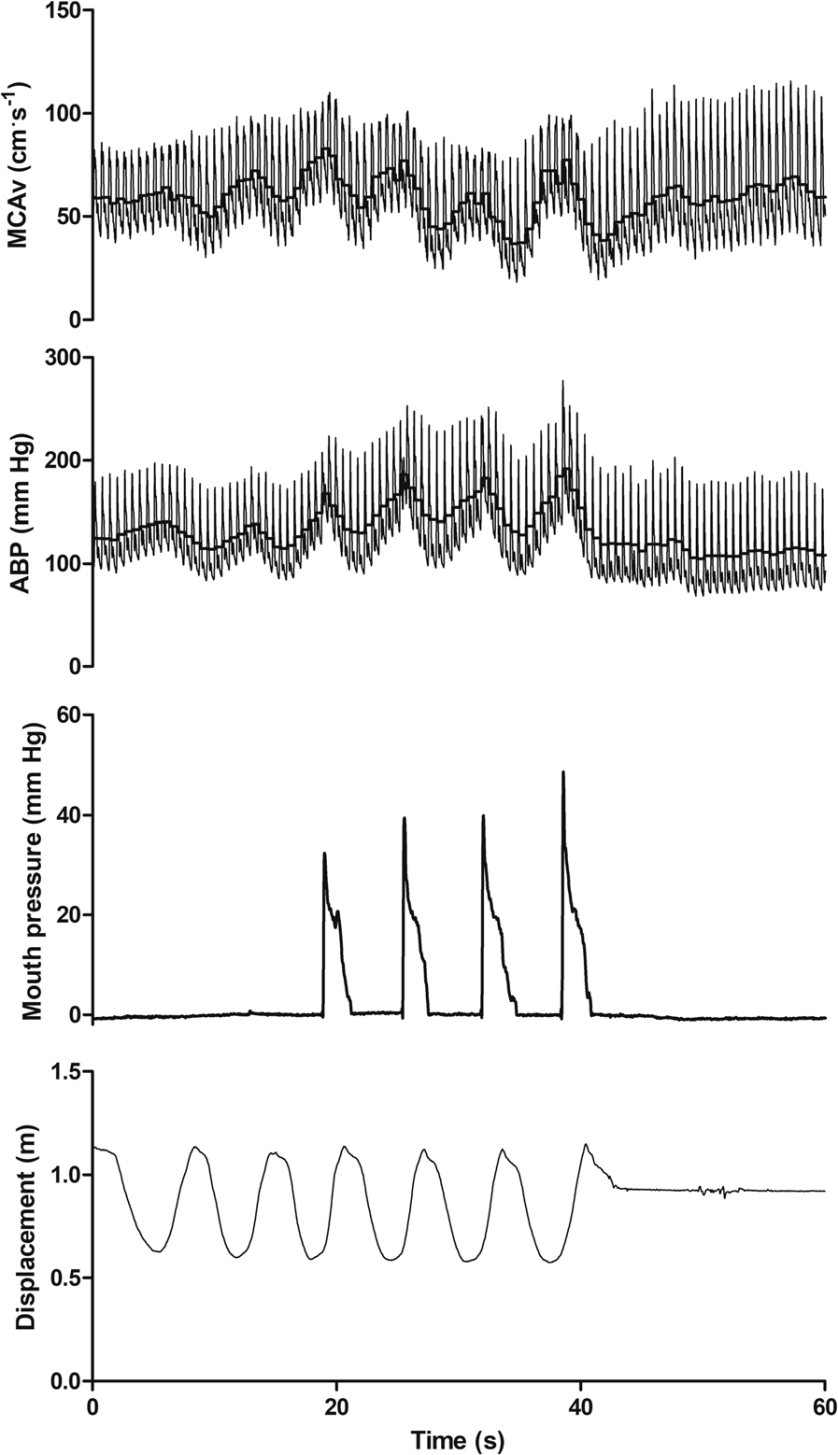
Haemodynamic response to 6 upright squats at 60% of one repetition maximum. MCAv middle cerebral artery blood velocity; ABP arterial blood pressure. The thick black line in the MCAv and ABP traces represents the mean for each cardiac cycle. Note that a reduction in displacement indicates the eccentric phase of the squat. A VM was only performed on the last 4 repetitions of the set, noting the resultant increase in MAP. Peak VM pressure occurs at the transition from eccentric to concentric contraction and coincides with peak blood pressure. Data from Perry et al. [55]

During RE the VM acts to stabilise the trunk, providing a mechanical advantage [39, 91]. However, the VM exacerbates the blood pressure perturbations during RE and is discouraged even in healthy adults [92]. For the advanced lifter, the manoeuvre is unavoidable at ≥80% of maximal voluntary contraction (MVC) or during repeated efforts when approaching fatigue [39]. As load increases the VM intensity, and therefore intrathoracic pressure, also increase [93] with more intense strains generating greater phase I MAP peaks [57]. The relative loading to specifically target power, strength, and hypertrophy may all exceed 80% of MVC during a training cycle [94]. As such, it is likely that resistance training individuals, irrespective of desired outcome, regularly perform a VM. During static RE with a VM, the haemodynamic profile is dominated by the VM [79], that is, all phases of the VM are discernible despite the background of sustained muscular contraction (Fig. 1). In contrast, as ~15 s of straining is required to discern all phases of the VM [42, 95], the short strains experienced during dynamic RE are masked by the larger exercise dependent MAP fluctuations [55]. Indeed, peak strain intensity, as indicated by oesophageal pressure (surrogate for intrathoracic pressure), occurs during the transition from the eccentric to concentric phase during dynamic RE and aligns with peak blood pressures [96] (also see Fig. 3). Therefore, understanding the haemodynamic profile during RE is dependent upon a complex interaction between RE type and VM recruitment.

## Physiological Response During Resistance Exercise

### Cardiovascular Response to Resistance Exercise

#### Blood Pressure

Despite the cerebral vasculature possessing well-developed autoregulatory mechanisms, the rate of change in CPP during dynamic RE provides a substantial challenge to blood flow regulation. The exact blood pressure response to RE is dependent upon contraction type and within the spectrum of RE, blood pressure responses vary greatly. Static RE produces modest, intensity dependent increases in blood pressure [97]. From contraction onset MAP steadily increases [98] to an eventual plateau, the beginning of which varies depending upon contraction time and intensity [79, 99, 100]. However, heavy dynamic RE produces extreme sinusoidal fluctuations in blood pressures (Fig. 3), with an individual peak of 480/350 mm Hg (systolic/diastolic) reported and group average of 320/250 mm Hg during dynamic bilateral leg press exercise [101]. Indeed, the extent of within-exercise hypertension is dictated by recruited muscle mass, with exercises utilising large muscle groups (e.g. leg press) generating the greatest hypertension [102–105]. Blood pressure responses are intensity dependent [105] and mediated by increases in both systolic and diastolic pressures [39, 106–108].

Within a dynamic movement, peak blood pressures are seen at the joint angle that corresponds with the weakest point of the strength curve [39, 109], which for leg press exercise corresponds with peak knee flexion [39] and generally coincides with the transition between eccentric and concentric contractions. Blood pressure declines as the concentric phase progresses [109] and the cycle repeats for each repetition (Fig. 3). Peak concentric blood pressure also increases in subsequent repetitions within a set [96, 101, 105], in subsequent sets of the same exercise [107], or if the rest period between sets is reduced [110]. Sale et al. [96] investigated the blood pressure response to bilateral leg press during various contraction types (concentric, isometric, and eccentric) using an isokinetic dynamometer and reported that irrespective of contraction type, the primary determinant of blood pressure was the voluntary effort, determined by force output rather than measurement of perceived exertion. Nevertheless, intense multi-joint efforts will evoke the greatest within-exercise blood pressure. Therefore, under the term RE, it is possible to generate disparate blood pressure profiles, dependent upon the type of contraction; i.e. static RE produces a relatively slow developing and modest increase in MAP that plateaus, whereas cyclic swings in blood pressure typify dynamic RE.

#### Cardiac Output

In comparison to the classification of blood pressure during RE, a paucity of cardiac output data is available, which is problematic for the description of CBF regulation given cardiac output modulates CBF independently of blood pressure [22]. During high-intensity leg press exercise (95% 1RM), SV is reduced and elevations in cardiac output are driven primarily by HR [109], with similar results found during static handgrip exercise [99, 111, 112]. However, this is not a consistent finding for isometric exercise during static leg extension with [79], and without [113], the VM. It would be expected that the VM would inhibit venous return such that HR does not compensate for the reduction in SV [79]. In contrast, unloaded upright squatting exercise increases preload and cardiac output [114, 115], and it should be noted dynamic contractions would facilitate venous return due to the skeletal muscle pump. As MAP undergoes cyclic changes throughout a single repetition, primary determinants of SV, such as afterload and preload will also change dynamically. Indeed, phase-dependent changes in cardiac output have been reported previously, with increases demonstrated throughout leg press exercise, peaking at the end of the concentric phase driven by a recovery of SV [109]. Nevertheless, during muscle contraction, irrespective of type, the prime driver for an increase in cardiac output appears to be heart rate.

### Cerebral Blood Flow Regulation During Resistance Exercise

As mentioned above, regulation of CBF is complex with stringent processes to ensure adequate perfusion, and ultimately, maintenance of function [116]. Exercise, of any nature, can challenge CBF regulatory mechanisms as multiple determinants of CBF may exert their effects simultaneously. Recently, Smith and Ainslie [30] have suggested that the current literature is unable to clearly identify the individual roles and contributions of all regulatory variables to the observed CBF responses and is indicative of the intricate-dependent relationship many variables demonstrate (e.g. P_a_CO_2_ and MAP). The majority of studies investigating the cerebrovascular response to exercise have utilised steady-state aerobic exercise, in particular upright or semi recumbent cycling [22, 117–126]. Even within the general classification of dynamic aerobic exercise some modalities, like rowing, demonstrate a markedly different CBF profile from cycling, with clear influence of blood pressure in the former. Indeed, during rowing MCAv profiles similar to dynamic RE are observed, sinusoidal, and often commensurate with blood pressure [127, 128]. The inherent complexity of CBF regulation is compounded by the VM and associated mechanical effects of perturbations in ICP during RE and rowing. Similarly, running produces rhythmic oscillations in blood pressure and MCAv [129], produced by interference between stride frequency and heart rate [130]. Whilst the hierarchy of cerebrovascular regulators still exists during RE (i.e. P_a_CO_2_ is the most potent [65]), it is evident that the cerebrovascular response is not uniform between exercise types. For instance, cardiac output contributes to MCAv elevations more during cycling than during rhythmic hand grip exercise [73]. Therefore, knowledge of cerebrovascular function during aerobic exercise cannot be extrapolated to RE.

Moreover, the different blood flow profiles between exercise modalities, including the various types of RE outlined within this review, may elicit different adaptive processes during habitual exposure (i.e. training). A key mechanism for exercise-related improvements in vascular function is attributed to the frictional forces that result from the mechanical movement of blood across the vascular endothelium (i.e. shear stress [131]). As outlined below, RE can provoke a wide range of blood flow responses during and following a bout, the consequences of which related to improved or impaired vascular function are largely unknown.

#### Dynamic RE

During RE a fourfold increase in arterial blood pressure during the concentric phase of lifting has been reported [101], greatly exceeding the traditionally proposed upper autoregulatory limit [31]. During high intensity cycling exercise dynamic cerebral autoregulation is active and able to effectively modulate CBF [126, 132, 133]; however, given the rapid nature of the fluctuations in blood pressure during dynamic RE, cerebral autoregulation is likely insufficient, such that MCAv varies commensurate with MAP [12, 65] as is observed during leg press [12] and squatting with additional load [55]. As impressive as the peak blood pressures appear, several authors indicate that ΔMAP dictates the cerebrovascular response [12, 63], illustrating the high-pass filter [134] characteristics of the cerebral circulation and reflecting the inherent latency (~5 s) of autoregulatory mechanisms [135]. The variation of MCAv with MAP persists during within-exercise hypertension, despite hysteresis [136–138]. Evidently, repeated body weight squatting manoeuvres have been used to perturb MAP and assess dynamic cerebral autoregulation [138, 139].

Habitual RE reduces central arterial compliance [140] and we have previously speculated that the cerebral circulation is not excluded from such adaptations. As cerebrovascular compliance modulates cerebral autoregulation [141], it is plausible that repeated hypertensive stimuli may modify autoregulatory function. We have shown that despite the substantial differences between exercise types, aerobically trained individuals (excluding rowing) and resistance trained individuals demonstrate similar dynamic autoregulatory capacity during forced MAP oscillations (repeated squat stands) [142]. However, we did demonstrate a trend for transfer function derived gain to be greater during slower frequency (0.05 Hz) oscillations in MAP in the resistance trained cohort. Moreover, resistance trained individuals demonstrate higher MCA pulsatility at rest [143], likely due to the reduction in pulsatile buffering capacity of the central arteries. These data indicate subtle modifications in cerebrovascular function; however, given the plasticity of cerebral autoregulation [144], it is possible that improved function in resistance trained individuals may be revealed at high perfusion pressures and/or possess a greater effective autoregulatory range.

Reports to date on the MCAv_mean_ response during dynamic RE (all exercises) are equivocal, with some reporting increases [63–65, 70, 76], no change [12] or a decrease [62]. Studies utilising leg press RE have reported all possibilities. It should also be considered that a simple average over the course of dynamic RE does not reflect the MCAv profile during exercise, as highlighted in Fig. 3. If leg press exercise is considered, studies that demonstrated an increase [64, 65] or no change [12] in MCAv_mean_ during exercise did not use a VM, but did use 80–100% of 10 repetition maximum. However, as the VM is recruited ≥80% of MVC or during repeated efforts when approaching fatigue [39], it is unlikely that the load used is a true reflection of the 80-100% 10 repetition maximum. In contrast, Dickerman et al. [62] reported that during a one-repetition maximum leg press exercise bout in elite level resistance trained individuals, the VM was responsible for a 25% reduction in MCAv_mean_. However, these authors did not report P_a_CO_2_ (or partial pressure of end tidal CO_2_ (P_ET_CO_2_) as a proxy). Similar to rest, P_a_CO_2_ exhibits a strong influence on CBF during RE [66], which is important given the typical pre-exercise breathing patterns during high intensity lifts comprises of hyperventilation [15], followed by an intense VM. Indeed, pre-exercise hyperventilation that reduced P_ET_CO_2_ by ~8 mm Hg (absolute value = 26 mm Hg) lowered exercise (leg press at 80% of 6 RM) MCA_mean_ by ~39% without a concurrent VM [65]. It is also worth noting that hypocapnia improves dynamic cerebral autoregulation [19]. Hyperventilation therefore may serve a complex role during RE, namely: (1) act as a pre-exercise routine, (2) reduce the absolute CBF (hyperperfusion) during RE, and (3) improve autoregulation such that changes in CPP are more adequately buffered.

In addition to hyperventilation the VM may also protect the cerebral vasculature during exercise via a reduction in transmural pressure [50]. The VM generates additional increases in MAP, and whilst initially this would appear detrimental, we have reported that peak MCAv_mean_ was similar between 90%, 60%, and 30% of 6RM, despite the greatest increase in MAP observed for the 90% set, with the VM only recruited at this intensity [55]. At the transition point between eccentric and concentric contraction, when VM recruitment is permissible, the greatest oesophageal pressures are observed, which subsequently decline as the concentric phase progresses in a remarkably similar fashion to MAP [96, 109]. As the VM is unavoidable at intensities ≥80% of MVC [39], it is plausible that participants self-select the VM strain intensity [intrathoracic pressure] to match exercise intensity, and therefore blood pressure; evident as when participants approach fatigue, which is associated with greater voluntary efforts, both MAP and strain intensity rise [96, 109]. Whilst speculative, this inherent VM self-selection intensity could culminate in proportionate increases in ICP, limiting CBF during peak CPP and mitigating drastic perturbations in cerebrovascular transmural pressure. As thoracic pressure and MAP decrease concurrently throughout the concentric phase, CPP is stabilised as both ICP and MAP would fall concurrently, maintaining CBF. Taken together, the anticipatory hypocapnia and use of the VM modify the CBF-blood pressure relationship during RE with the latter potentially providing a temporary protective mechanism.

#### Static Resistance Exercise

During static RE (hand grip and knee extension), increases in MCAv_mean_ [22, 71, 72, 74, 76, 79], internal carotid artery [67, 71] and vertebral artery [81, 82] blood flow have been reported. However, some authors have reported no change in MCAv_mean_ during static hand grip [66] or leg extension [75]. Similarly to dynamic RE, MCAv_mean_ is dependent upon the ventilatory response and subsequently P_a_CO_2_, with pre- [145] and within- [72] exercise hyperventilation lowering exercise and recovery MCAv_mean_. Braz et al. [66] reported that during static hand grip exercise, MCAv_mean_ elevations were evident only when P_ET_CO_2_ was clamped 1 mm Hg above resting. Therefore, discrepancies may result from an overriding influence of hypocanpic vasoconstriction and corroborate findings in dynamic RE.

Dynamic cerebral autoregulation has been reported to be unaffected during and following static [78] and rhythmic handgrip [76] exercise. Static RE is associated with a prolonged pressor response with a plateau in MAP [45, 71, 79]. Hirasawa et al. [71] reported a plateau in ICA flow 60 s into a 120-s unilateral static leg contraction at 30% of MVC. External carotid artery blood flow, however, demonstrated a continual rise, concurrent with small increases in MAP in the later stages, indicative of a potentially protective mechanism by directing flow extracranially during hypertension [83]. Whether this persists during dynamic exercise with larger increases in MAP is unknown, and difficult to assess with current imaging methodologies (see Measurement challenges and considerations). Using maximal static bilateral leg extension (without VM), Pott [79] reported a sharp increase in MCAv (~10 cm^.^s^-1^), peaking at ~3 s but returning to baseline by ~5 s. Moderate increases in PCAv (~6 cm^.^s^-1^) have also been shown in static handgrip exercise at 30% of MVC [146]. Further studies that did not report a time effect have also reported increases in MCAv [78], PCAv [81], ICA [67] and VA flow [81]. Interestingly, we have shown larger initial (time aligned with VM phase I) increases in MCAv, but not for PCAv, during RE with normal ventilation compared to exercise with the VM [45]. However, these effects diminished with contraction time such that no differences were observed after ~12s, likely reflecting the engagement of cerebral autoregulation after >5 s such that blood velocity is stabilised [135].

It should also be considered that during single limb exercise the alterations in blood flow to the brain are heterogeneous. Firstly, when considering cerebrovascular autoregulation some [147, 148], but not all [149], report regional differences in blood flow responses to blood pressure perturbations. Similarly, cerebrovascular reactivity to CO_2_ [150, 151] may differ between the posterior and anterior circulations, although this is not a universal finding during hypercapnia [21]. Secondly, more pronounced elevations in blood flow are observed in vessels contralateral to the exercising limb due to the anatomical location of motor areas within the brain, evident during unilateral RE. For example, Linkis et al. [77] reported that right hand contractions produced a 19% increase in contralateral MCAv with no change in ipsilateral MCAv. Increases in blood flow to premotor and motor sensory areas have been reported during low-intensity static handgrip [68] and leg extension [152], with no change in global hemispheric flow as measured using the xenon clearance technique. Increase in blood flow is mediated by NVC and has been observed in the contralateral ICA [67] and VA [81], and supported by increases in MCAv [70, 73] during unilateral static exercise. Ipsilateral ICA blood flow appears to be restrained by sympathetic vasoconstriction during unilateral hand grip exercise [67]. In support of autonomic regulation during RE, intracranial arteries such as the MCA undergo sympathetic vasoconstriction during rhythmic hand grip exercise [59]. Importantly, the blood flow response in the contralateral hemisphere is dependent upon volitional contractions (central command) as passive rhythmic exercise and electrically stimulated exercise do not alter blood velocity in the anterior cerebral artery [80]. Due to the slow and progressive increase in MAP during static RE without a VM the effect of NVC can be observed, in contrast to dynamic RE where this response is masked by rapid changes in CPP. Irrespective of the nature of contraction, the increase in CPP may exacerbate NVC mediated increases in cerebral perfusion. Whilst the exact role of the autonomic nervous system during RE is yet to be elucidated, the primary driving factor of CBF during dynamic RE appears to be CPP; conversely, during low-intensity static contractions NVC are likely the primary driver. It appears that the slow progressive increases in CPP during static RE can be effectively countered by cerebral autoregulation. Therefore, application of findings in small muscle groups utilising static contractions to all types of RE should be done with caution.

## Physiological Response Following Resistance Exercise

### Blood pressure immediately following resistance exercise

Reductions in mean, systolic, and diastolic blood pressures have been consistently reported immediately (within seconds) following RE [15, 64, 65, 153–155], with an increase in pressure pulsatility [156]. During muscle contraction blood flow is inhibited, with increases in intramuscular pressures occluding arterial and/or venous blood flow [157]. Following exercise, however, blood flow to active musculature increases [158] mediated by metabolic dilation; a functional hyperaemia [159, 160] that is aided by a reduction in transmural pressure during relaxation [161]. Hyperaemia following lower limb exercise is exacerbated in the upright position [162] and is dependent on contraction intensity [163] and frequency [164]. In support of this, we have previously observed a load dependent hypotension immediately following upright squatting with the greatest relative intensity producing lower absolute blood pressures [55]. Moreover, MAP time to recovery and time below baseline were greater, mediated by acute reductions in cardiac output and total peripheral resistance, with similar results observed following upright static deadlifting [165]. To date, studies investigating the haemodynamic response to RE have typically utilised a leg-press type movement [12, 64, 65], static/isometric type exercise [78, 103] or both [96, 102]. The leg-press position facilitates venous drainage, as the feet are at or above heart level, such that hypotension is not observed when participants remain semi-recumbent [12]. Thus, the upright position in combination with greater exercise intensities exacerbates the transient post-exercise hypotension irrespective of contraction type. Similarities of post exercise hypotension following RE can occur with initial orthostatic intolerance, with the latter characterised by venous pooling [166], and reductions in venous pressure [167] and total peripheral resistance [168]; this culminates in a rapid translocation of the blood into the limbs with the abrupt reduction in MAP exceeding the baroreflex control of vascular tone [169]. Considering these mechanisms in the context of post RE hypotension, it is likely that functional hyperaemia to musculature below heart level and the rapid reduction in intramuscular pressure would exacerbate these responses in an intensity dependent manner.

### Cerebrovascular Response Following Resistance Exercise

Cerebral hypoperfusion sufficient to induce syncope has been reported immediately following RE [15] and is likely driven by the hypotension described above. However, pre-exercise hyperventilation lowers pre- and within-RE MCAv [65] likely increasing the risk of post-exercise syncope. Despite hypocapnia improving autoregulatory capacity, the speed of hypotension onset outpaces the reflex cardiovascular and cerebrovascular countermeasures such that decrements in CBF persist. In the absence of P_a_CO_2_ perturbations, cerebral autoregulatory phase is reduced immediately following dynamic RE, indicating an impaired regulatory capacity [63]. The magnitude of CBF reduction is dependent upon the load lifted when upright and closely aligns with the MAP pattern following exercise cessation, with the largest, and most prolonged, decrease in MCAv observed after lifting the highest relative load [55]. Importantly, the highest relative load coincided with the recruitment of the VM, the implications of which are discussed in the following section. Without the presence of hypotension, MCAv declines below baseline following lower limb RE when in a semi-recumbent position emphasising that ΔMAP, not absolute MAP, drives the CBF response [12]. Notwithstanding, recent evidence indicates dynamic autoregulation is modified by body position with reduced function during standing [86], compounding the effect of greater hypotension following upright exercise. Of note, the decrement in MCAv following exercise is primarily due to reductions in diastolic blood velocity [55], similar to pre-syncope [170]. However, the increase in pulsatility is acute, with normalisation apparent 10 min following exercise cessation [156]. As with the responses during exercise, the post RE cerebrovascular responses still appear to be dominated by the rapidly changing CPP. Whilst hypotension is not always observed following RE due to body position, hypotension is associated with greater reductions in cerebral perfusion and the recruitment of the VM.

### The Role of the Valsalva Manoeuvre Following Resistance Exercise

**Fig. 4:**
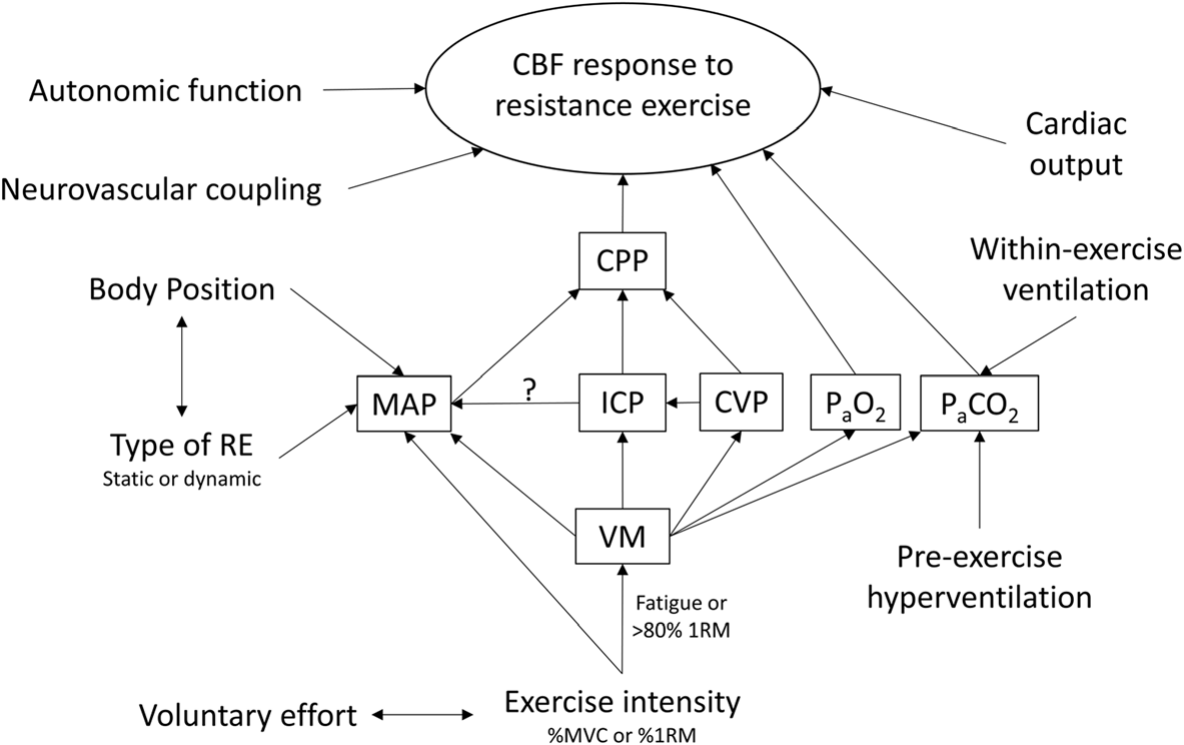
Summary of the physiological regulators of cerebral blood flow (CBF) during RE. RE resistance exercise, CPP cerebral perfusion pressure, MAP mean arterial blood pressure, ICP intracranial pressure, CVP central venous pressure, P_a_CO_2_ the partial pressure of arterial carbon dioxide, P_a_O_2_ the partial pressure of arterial oxygen, VM Valsalva manoeuvre, MVC maximal voluntary contraction and 1RM one repetition maximum

The role of the VM during RE is complex (Fig. 4), with phase-dependent effects apparent. As mentioned previously, the effect of this VM may limit cerebral perfusion *during* exercise and be viewed as beneficial; however, *following* exercise, the detrimental circulatory effects of the VM are apparent. Short duration VMs (~10s) in isolation produce phase II reductions in cerebral oxygenation (Fig. 2) [57], sufficient to induce syncope when standing [47]. Therefore, it is not surprising that syncope is has been reported following upright high intensity RE when the VM is recruited [15]. In the upright position, the combination of the release of the strain, exercise-induced functional hyperaemia to the dependent musculature below heart level (e.g. during Olympic style lifts, squatting or deadlifts), and pre-exercise hyperventilation threaten maintenance of CBF following RE [55, 65]. Whilst pre- and within-exercise hyperventilation likely contribute to post-exercise cerebral hypoperfusion, the rapid reduction in CPP likely underpins post exercise syncope, as pre-straining hyperventilation is not present in syncope following even short VMs [47]. As VM-phase III hypotension and MCAv reductions are less when supine [42] or seated (Fig. 1), the likelihood of post-exercise syncope is likely mitigated in these positions.

Residual cerebral vasoconstriction, mediated by hypocapnia, cerebral autoregulation, or both, likely persists following exercise due to inherent latency of cerebrovascular reactivity to CO_2_ [171] and autoregulation [135]. As such, CPP may rapidly decline whilst the cerebral vasculature is constricted, although this would depend on the length and timing of the release of the strain. Long straining periods, like those observed in complex, multi-phase lifts (Olympic style lifting) [15], likely exacerbate the issue, as cerebral perfusion is depressed for longer periods and oxygenation compromised. Therefore, the effects of body position and the VM act in concert to compound the haemodynamic stress immediately following RE. Other interactive possibilities also exist during dynamic RE and require further investigation—for example (1) a single VM may span several repetitions, and (2) the timing of a brief VM may generate a scenario where the release of the breath hold during late phase I (MAP declining) coincides with the cessation of exercise and associated functional hyperaemia, such that a rapid decline in MAP ensues despite a short straining period.

Hitherto, we have focused on the most common post-exercise clinical presentation—syncope. Whilst syncope is the obvious adverse post-exercise event, a greater potential issue is the prevailing cerebral hyperperfusion. With the VM alone, we have previously reported a near three-fold increase in phase IV MCAv from baseline, where the greatest peak in MCAv was achieved (see Fig. 2 for across phase comparison) [47]. During static RE, this response persists (Fig. 1), with greater peak blood velocities >3 s after exercise associated with elevated pulsatility in the PCA and MCA [45]. Similarly, seconds following dynamic RE with a VM, rapid increases in cerebrovascular conductance and MCAv are evident [55]. Further investigation is required to quantify the acute and long-term implications of repeated VMs during RE to inform safe practice, although several methodological challenges must be overcome first.

## Measurement Challenges and Considerations

Investigating RE presents many challenges, primarily methodological limitations that include measurement of blood pressure. Non-invasive (e.g. finger photoplethysmography) and direct arterial measurement [101] of blood pressure require one hand to be free and resting, limiting the scope of exercise to either lower limb, or unilateral upper limb RE. Thus, appropriate bilateral blood pressure comparisons between upper and lower limb exercise are missing which inhibits exploring potential differences in CBF-blood pressure relationships between different exercises and/or muscle groups. Another inhibitor of physiological measurement is the physical movement of the participant during RE, limiting most research to static RE. Given the majority of RE performed [94] and recommended [10, 92, 172, 173] is of the dynamic variety, issues arise with extrapolating findings to all variants of RE. However, the greatest challenge is measuring CBF, as discussed below.

The accuracy of transcranial Doppler has recently been questioned [69, 174] during large changes in blood pressure [59] and P_a_CO_2_ [88, 175, 176], conditions that typify RE. Inferences of blood flow from velocity should be made with caution [174] as the use of transcranial Doppler ultrasound as a surrogate for blood flow requires a stable arterial diameter. Using magnetic resonance imaging, rhythmic handgrip exercise (60% MVC) produced a 2% decrease in contralateral MCA surface area, suggested to be a direct result of sympathetic vasoconstriction [59]. As changes in MCA diameter occur during moderate intensity exercise with a small unilateral muscle mass (hand grip exercise), the constancy of intracranial arterial diameter during RE (dynamic or static) with a large muscle mass cannot be assumed. Additionally, during transient hypotension blood velocity recordings may underestimate flow, particularly in the posterior circulation [149]. Further investigations are required to establish whether sympathetically mediated vasoconstriction persists at high-intensity dynamic RE or if cerebral arteries are passively dilated by the extreme intramural pressure (MAP >300 mm Hg) [101]. As such, the data utilising transcranial Doppler must be interpreted with caution.

Given the uncertainty surrounding the constancy of intracranial arterial diameter, researchers commonly use duplex ultrasound to measure blood flow in upstream extracranial feed arteries to corroborate transcranial Doppler data [177]. However, participant movement largely excludes this technique and others (e.g. magnetic resonance imaging) during dynamic RE. Moreover, temporal resolution sufficient to capture beat-to-beat flow is required to encapsulate the dynamic nature of the haemodynamic profile of RE. Imaging transcranial doppler devices could provide a solution; however, measurement of intracranial vessel diameter remains an issue [178]. In addition to complicating the haemodynamic profile, the VM complicates the measurements of CBF and is largely avoided [12, 63–65, 67, 78, 81] (Tables 1 and 2). During the VM, the internal jugular vein fills with blood, obfuscating the common carotid artery and its bifurcations, limiting the measurement of blood flow to the vertebral arteries. Whilst possible to obtain ICA blood flow during a VM, longer VMs are required (>30s), increasing the likelihood of adverse advents (e.g. syncope) and reducing applicability. Nevertheless, ongoing investigations into cerebrovascular control during RE with a concomitant VM are pertinent, as persons training at high intensities, or to near fatigue, will likely utilise the VM. The anatomy of the brain and skull remains an issue for the researcher, particularly when quantifying CBF. These issues are not new [179]; however, they preclude the accurate measurement of intracranial blood flow with sufficient temporal resolution and accuracy to reveal a more comprehensive understanding of CBF regulation during RE.

## Conclusions

Under the blanket term of exercise disparate blood pressure and CBF responses exist. Further, within RE alone, the type of contraction generates markedly different CBF responses. Given these clear differences, it is plausible to expect the relative contributions of regulatory mechanisms to the observed CBF response to differ in contribution between types of exercise, and the findings from one type cannot be applied ubiquitously. It appears that the hierarchy of CBF regulation still exists during RE, with P_a_CO_2_ dominant. However, sinusoidal perturbations in MAP produced by dynamic RE and the inclusion of the VM profoundly challenge cerebrovascular regulation, with the latter dominant during a background of static RE. Further research is required, particularly during dynamic resistance exercise, to (1) elucidate the contribution of the VM to CBF regulation, (2) quantify the role of the sympathetic nervous system, (3) establish more accurate means of measuring CBF during large changes in MAP, and (4) establish the chronic cerebrovascular adaptations to RE. The effectiveness of RE cannot be ignored; however, a more complete understanding of haemodynamic responses is required to inform safe practice.

## Data Availability

Data sharing not applicable to this article as no datasets were generated or analysed during the current study.
